# Stability of Film-Forming Dispersions: Affects the Morphology and Optical Properties of Polymeric Films

**DOI:** 10.3390/polym13091464

**Published:** 2021-05-01

**Authors:** Maria Gabriela De Paola, Rosy Paletta, Catia Giovanna Lopresto, Giuseppe Emanuele Lio, Antonio De Luca, Sudip Chakraborty, Vincenza Calabrò

**Affiliations:** 1 Department of Computer Engineering, Modeling, Electronics and Systems (D.I.M.E.S), University of Calabria, I-87036 Rende, Italy; mgdep66@gmail.com (M.G.D.P.); rosypaletta9321@gmail.com (R.P.); vincenza.calabro@unical.it (V.C.); 2 Department of Physics, University of Calabria, and CNR-Nanotec, I-87036 Rende, Italy; giuseppe.lio@unical.it (G.E.L.); antonio.deluca@fis.unical.it (A.D.L.)

**Keywords:** starch-based films, carboxymethyl cellulose, film-forming dispersions, Turbiscan, characterization, optical properties

## Abstract

Starch-based films are promising alternatives to synthetic films in food packaging. They were widely studied in terms of mechanical and optical properties. In food packaging, optical properties are of great interest because ultra violet (UV-light) protection is strictly required. Nevertheless, the characterization of film-forming dispersions was poorly addressed, especially regarding its correlation with the film produced. In this work, we characterized film-forming dispersions at different compositions of starch and carboxymethyl cellulose (CMC) by Turbiscan. This instrument is based on multiple light scattering and gives significant information about the miscibility of polymers in the dispersed phase. Indeed, it identifies the phenomena of destabilization and phase separation before their visibility to the unaided eye. This work aimed to study whether the homogeneous/inhomogeneous morphology of films could be forecast by the analysis of profiles obtained in the dispersed phase. The films produced were investigated by optical microscopy and absorbance analysis. As the CMC fraction increased, Turbiscan showed reduced phase separation. This implies better miscibility of mixture components and higher gelification degree. The related film was more homogeneous and presented higher UV absorbance. Consequently, film-forming dispersions and optical properties of films are strictly correlated and Turbiscan-based analysis is very useful to investigate the dispersion stability and predict the film quality.

## 1. Introduction

Despite their excellent mechanical properties, fossil-derived plastic materials cause environmental pollution due to their non-biodegradability. Therefore, there is a growing interest in replacing them with compostable and non-polluting biopolymers, moreover in food packaging [1]. There are many other different polymers used for film formation for various industrial applications around the world [2,3], either in batch or in immobilized condition. Some polymeric membrane has also been used for recovery of reactive solvents or chemicals used in complex aqueous solutions [4,5]. Biopolymers can be produced from abundant, cheap, and renewable sources, and are good barriers for oxygen and carbon dioxide. The most studied biopolymers are carbohydrates, particularly starch.

Starch-based films are brittle, so plasticizers (e.g., glycerol) are usually used to enhance their flexibility [6]. Interactions among polymeric chains are needed to form films and are promoted by the addition of cross-linkers. Citric acid is commonly used as a cross-linker forming interactions between its carboxyl groups and hydroxyl groups in starch [7]. Citric acid strengthens the film structure, but it does not increase the film water permeability [8]. Starch-based films are generally prepared by the wet method. Starch and additives are dissolved in hot water to form a dispersion. This dispersion is poured and dried in a Petri dish or Teflon, until film formation occurs.

More interesting properties can be observed by introducing high molecular weight heteropolysaccharides into the dispersion, to improve mechanical properties and humidity control. Among them, carboxymethyl cellulose (CMC) has proved to be very promising. It is a non-toxic and non-allergenic ether cellulose and it can bind water, thanks to the high number of carboxyl and hydroxyl groups. CMC has a high viscosity and its chemical structure, which is similar to starch, limits miscibility problems in the initial dispersion [9]. The observable effects on films are the increase of tensile strength and the decrease of elongation [10].

Optical properties also are relevant for food packaging. Sometimes, a high degree of transparency is preferred for better visualization, but opacity helps to increase shelf life, protecting against incident light [11].

Generally, films are well characterized from a mechanical and optical point of view [12], but less attention is paid to characterizing the film-forming dispersions by the study of rheological properties [13] and stability. In literature, stability is evaluated in terms of zeta potential [14], because charges of polymer chains affect the kind of aggregation and, consequently, the microstructural network [15].

Turbiscan allows an in-depth characterization of film-forming dispersions, by visualization of the main phenomena of particle migration (sedimentation, creaming) and aggregation (flocculation, coalescence). This instrument, based on multiple light scattering, can calculate a stability index of dispersions (TSI, Turbiscan Stability Index) by comparing backscattering and transmission profiles during the time.

The Turbiscan equipment was used to investigate destabilization phenomena of inorganic phase change materials [16,17,18], but it is promising also in other dispersion-based applications.

When a film is formed from a polymeric dispersion, the first step of suspended particles coalescence is observed, followed by interactions among chains [15,19]. This starts the gelification process leading to a more stable dispersion, as observed in nano silica gelification [20].

The present work aims to study whether and how the characteristics of film-forming dispersions affect the morphology and optical properties of the films produced. Generally, the miscibility of polymers was studied on solid-state films by Fourier-transform infrared spectroscopy (FT-IR) analysis, X-ray diffraction, differential scanning calorimetry, light optical microscopy, and scanning electron microscopy [21]. Nevertheless, to the best of our knowledge, this correlation between the dispersion and the film produced has been never investigated in the literature.

Samples of three film-forming dispersions, based on starch-CMC, were characterized by Turbiscan, in terms of stability and destabilization phenomena to identify possible phase separation and evaluate the degree of miscibility among components already in the dispersed phase. Films were prepared from each dispersion by the wet method. Drying conditions were kept constant. The morphology of the obtained films was analyzed by optical microscope and the results were processed using Image J to define the degree of homogeneity.

Furthermore, light absorption tests were carried out on films in the UV area. Films used for food preservation, require no absorption in the UV area, especially in the case of foods with a significant amount of fats, because UV absorption is responsible for oxidative degradation [22].

## 2. Materials and Methods

### 2.1. Materials

Starch from potato and carboxymethyl cellulose CMC in different ratios was used as the main component for film preparation; sodium hexametaphosphate 65–70% P_2_O_5_ basis, glycerol, citric acid, and distilled water were also used in different ratios. All chemicals were supplied by Sigma-Aldrich.

### 2.2. Methods

#### 2.2.1. Dispersion and Film Preparation

Dispersions were prepared by the modified Ghanbarzadeh method [9]. Sodium hexametaphosphate is a non-toxic additive used in the food industry and it was added as a thickener. Glycerol and citric acid were added as plasticizer and cross-linker, respectively.

Three dispersions, named S, were prepared by stirring 5 g of starch, 2 mL of glycerol, 1 g of sodium hexametaphosphate, and 0.5 g of citric acid in 100 mL distilled water, firstly at room temperature for 5 min and then in a hot water bath at 90 °C for 30 min.

Then, three different CMC-based dispersions were prepared by stirring components at 75 °C for 15 min. The three dispersions named CMC_1_, CMC_2_, and CMC_3_ were prepared by adding, respectively, 0.5, 0.75, and 1 g of CMC in 75 mL of distilled water.

Finally, three dispersions were obtained by mixing two dispersions, one based on starch (the dispersion S) and another one based on CMC, as described in Table 1.

Dispersions S-CMC_1_, S-CMC_2_, and S-CMC_3_ were cooled to 40 °C. From each of them, 25 mL were taken for Turbiscan analysis, and 70 mL were taken for the film preparation.

Films were prepared according to the thermal-induced phase separation (TIPS). An amount of 70 mL of each dispersion was poured onto a Petri dish (diameter 9 cm, height 3 cm) and placed in a forced ventilation oven at 60 °C for 8 h.

#### 2.2.2. Characterization of Dispersions

**Figure 1:**
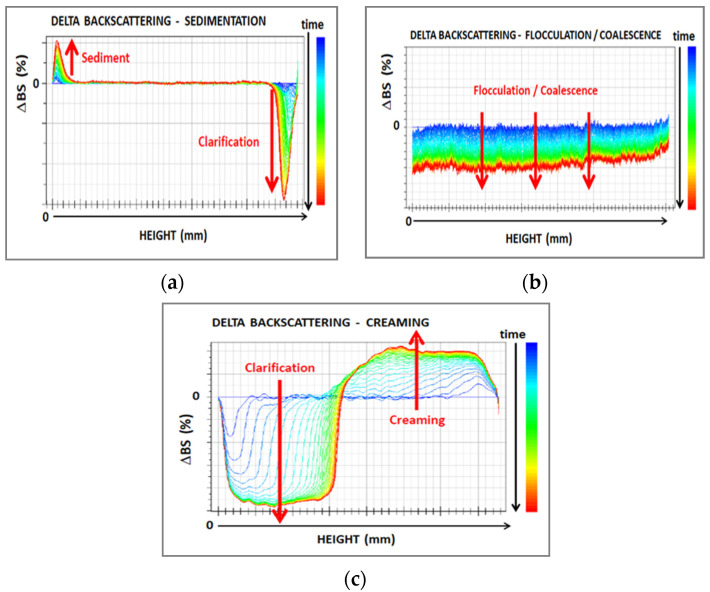
Main phenomena of destabilization in dispersions observed by Turbiscan analysis: (**a**) sedimentation; (**b**) flocculation/coalescence; and (**c**) creaming. (adapted from [16])**.**

The film-forming dispersions were characterized by Turbiscan (developed by Formulation, Smart Scientific Analysis) at 60 °C for 8 h. The Turbiscan equipment is based on multiple light scattering to identify the particles’ migration and their size change in liquid dispersions. It is provided with two detectors, working in transmission (T) and backscattering (BS) modes. This makes possible the analysis of both transparent and opaque samples. When T is higher than 0.2% the instrument works in transmission mode, whereas a value of T lower than 0.2% indicates backscattering mode. The “Delta” mode facilitates the visualization of destabilization phenomena, by using the first scan as a reference and by amplifying variations. Results were compared with reference ΔBS profiles in the whole cell shown in Figure 1 and adapted from the instrument handbook.

Therefore, Turbiscan identifies possible coalescence phenomena before gelification. Besides, the formation of a gelified structure can be correlated with the system stabilization [20], by the variation of the Turbiscan stability index (TSI). The TSI value is calculated by the instrument by comparing each scan to the previous one, at a selected height, and dividing the result by the total height selected (Equation (1))

(1) $$\mathrm{TSI}=\underset{I}{\sum }\frac{\sum_{h}|{\mathrm{scan}}_{i\;}\left(h\right)-{\;\mathrm{scan}}_{i-1\;}\left(h\right)|}{H}$$

The gelification degree is higher when the slop of the TSI vs. time curve decreases, because the system stability increases.

#### 2.2.3. Film Characterization

Film thickness—The thickness of films was measured with a digital Borletti caliper, taking measurements in several places and calculating the average value.

Morphology—Samples were analyzed by optical microscope Leica DMRX (Zeiss) to characterize the film morphology. Magnification power used in the examination was 50×. The microscope uses a white lamp in the entire visible range and the operative mode used was the reflection one. The microscope is equipped with a Canon 600D reflex camera with proper lens and mount for microscopic investigation. The scanned area has a total size of 40 μm × 100 μm, depending on the focal beam waist and charge-coupled device (CCD) sensor.

Light transmittance and opacity value—The absorbance analysis of samples was carried out by a dedicated spectroscopic system able to collect the transmitted signal through each sample. A Xenon lamp was used to produce white light radiation in a range from 250 to 1100 nm; then, the sample was mounted on a holder that permits having three-axis movement, whereas two optical fibers, coupled with two GRIN (graded-index) lenses, are used to focus the white light on the sample and to collect the transmitted part of the signal. A spectrometer (Ocean Optics, USB-4000), working in a wavelength range from 250 to 1100 nm), was used to measure the absorbed light, and returned its spectral behavior.

The “reference” signal in the spectroscopic measurements is acquired as a ratio between the white light of the Xenon lamp (shutter off) and the background signal (shutter on).

Besides, the film opacity was evaluated to ensure and validate the possibility of using films as a shelter from light (UV-Vis-NIR). Opacity is directly related to the intensity of light absorbed by the film at a particular wavelength. Opacity was calculated by dividing the absorption value at 600 nm by the film thickness according to [23] (Equation (2)).

Opacity (O) = (absorbance (λ = 600 nm))/(thickness (mm))(2)

## 3. Results and Discussion

### 3.1. Dispersions Characterization

Since the transmission signal (T%) was higher than 0.2% for all dispersion, the backscattering signal was not analyzed because it was due to internal reflections in cells [24,25].

**Figure 2:**
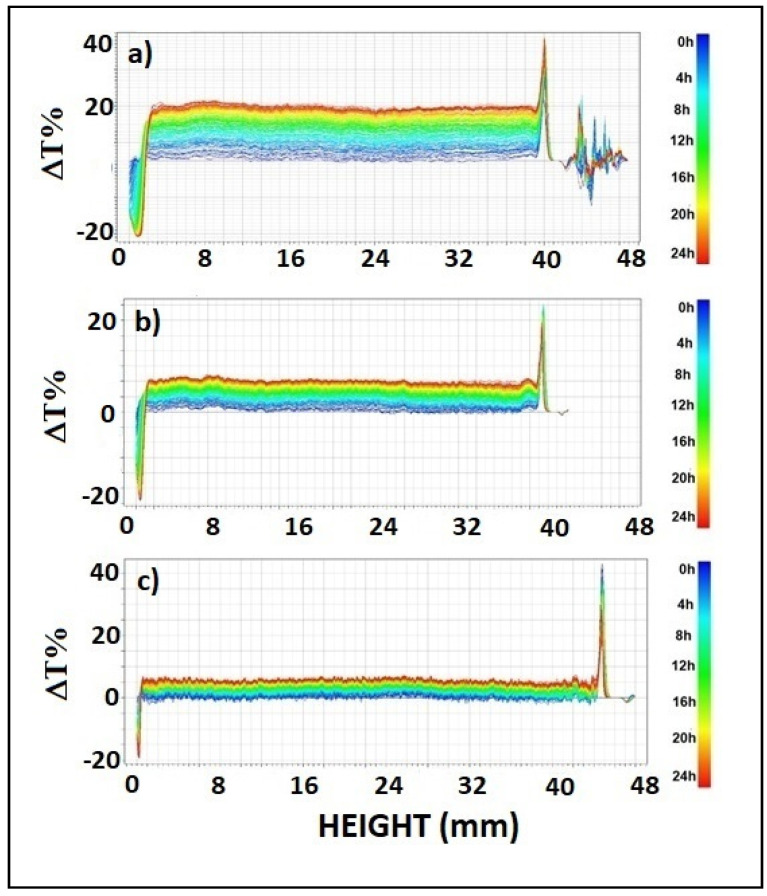
Profiles of Delta transmittance, ΔT(%), in each dispersion sample (analysis time: 24 h) (**a**) S-CMC_1_, (**b**) S-CMC_2_, and (**c**) S-CMC_3_.

Data were reported in ΔT% mode to better visualize destabilization phenomena. It is evident in Figure 2 that transmission profiles shifted upwards over the whole length of samples. Therefore, coalescence was the main destabilization phenomenon and particle aggregates were formed during the analysis time. Peaks visible at the top and bottom of samples indicated the presence of creaming and sedimentation, respectively. Increasing CMC content led to reduced coalescence. Indeed, the ΔT% values of samples of S-CMC_1_, S-CMC_2_ and S-CMC_3_ are 15, 10, and 5, respectively. Moreover, the peak width at the top and bottom of samples decreased when the amount of CMC increased, so that minor phase separation occurred.

The extent of phase separation was 6.58%, 5.43%, and 3.32% (in terms of volume) in S-CMC_1_, S-CMC_2_, and S-CMC_3_, respectively. It is associated with the mutual solubility of components. Even small phase separation could affect the film quality by causing irregular portions. This was less evident in a film obtained from dispersion S-CMC_3_. Probably, the mixture starch + CMC + additives was not soluble at every composition.

**Figure 3:**
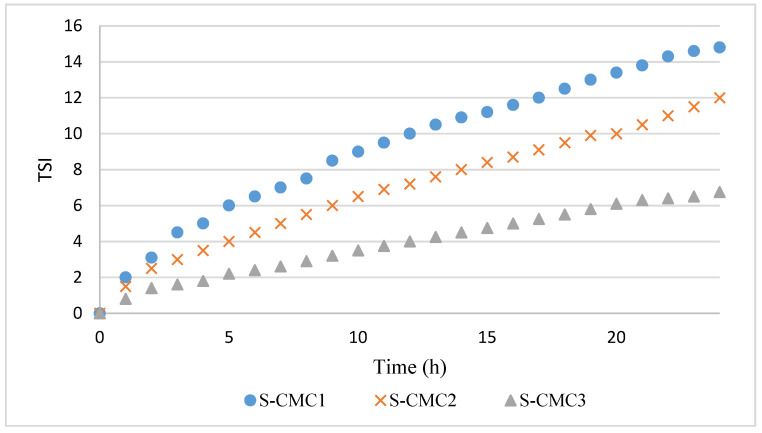
Profiles of Turbiscan stability index (TSI) over time for dispersions S-CMC_1_, S-CMC_2_, and S-CMC_3_.

The profiles of TSI over time are shown in Figure 3. The data were highly reproducible, with a negligible standard deviation.

The TSI profiles are coherent with ΔT%. Indeed, the dispersion S-CM3 has the lowest phase separation corresponding to the lowest TSI values during the analysis.

In all curves, a significant variation of the slope was observed during the first 2 h, corresponding with the first coalescence phase [16,20,26,27,28]. This slope change could be due to gelification beginning, when interactions among particles are formed before the film formation. After 2 h, the dispersion S-CMC_3_ presented the lowest slope, because of its higher stability and gelification degree.

The dispersion S-CMC_1_ presented the widest sedimentation peak and the highest slope of TSI in the second phase. The width of sedimentation peak and slope of TSI decreased in dispersion S-CMC_2_ and, further, in dispersion S-CMC_3_. Systems are more stable with increasing CMC content. Probably, increasing CMC concentration led to higher mixture viscosity and reduced mobility of suspended particles.

### 3.2. Film Characterization

#### 3.2.1. Films Thickness

The average value of film thickness, measured by a Borletti caliper, was equal to 0.35 ± 0.01 mm for the S-CMC_1_ film and 0.36 ± 0.01 mm for both S-CMC_2_ and S-CMC_3_ films.

#### 3.2.2. Optical Microscope Analysis of Films

**Figure 4:**
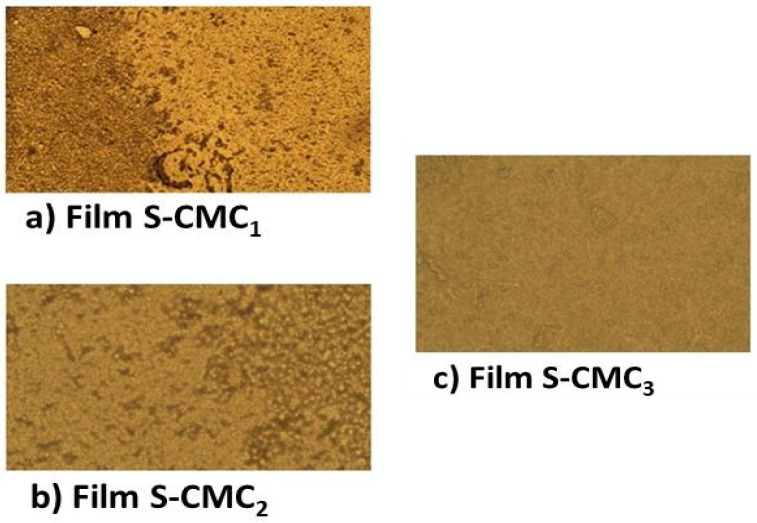
The micrographs show the surface morphology of the three produced films: (**a**) S-CMC_1_; (**b**) S-CMC_2_; and (**c**) S-CMC_3_. The images have been collected using a Zeiss microscope in reflection mode using an input objective lens with a magnification of 50×. Each image shows a 40 × 100 μm^2^ portion of film sample.

The optical microscope analysis, carried out by a Zeiss microscope, gave information about the sample morphology and revealed a progressive increase of homogeneity from sample S-CMC_1_ to sample S-CMC_3_, as evident in Figure 4.

S-CMC_1_ dispersion had the most evident phase separation and the highest variation of TSI; this affects the film produced, which was the most inhomogeneous in the microscopy analysis. Instead, S-CMC_3_ had no significant phase separation and the lowest variation of TSI, producing a homogeneous film. S-CMC_2_ dispersion and the film obtained from it had intermediate behavior. The optical measurements provide qualitative information about the roughness of the sample surfaces. It is noticeable that the S-CMC_3_ sample presented a surface that is almost flat without any inhomogeneity.

**Figure 5:**
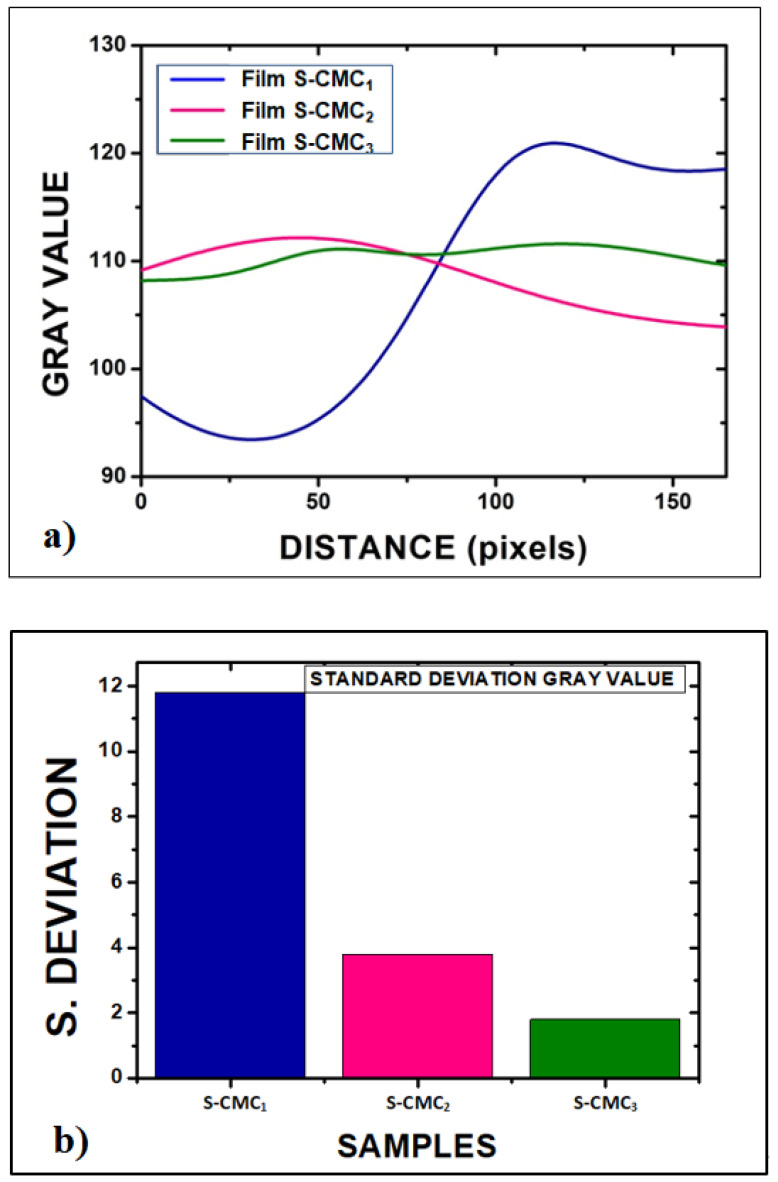
Quantitative assessment of the inhomogeneity degree of films: (**a**) Plot of the average of each sample profile function, after the optical image conversion into grayscale images by ImageJ software; (**b**) standard deviation of grey values related to inhomogeneity of films (11.8 for S-CMC_1_; 3.8 for S-CMC_2_; and 1.8 for S-CMC_3_).

For a quantitative assessment of the inhomogeneity degree, open-source software for image processing (ImageJ) was used to obtain a full morphological characterization. Each picture was converted into grayscale. Then, an average of each surface profile was extracted by using the plot profile function, as reported in Figure 5a. Finally, the standard deviation of grey values was related to the inhomogeneity of films, as shown in Figure 5b.

#### 3.2.3. Optical Properties

**Figure 6:**
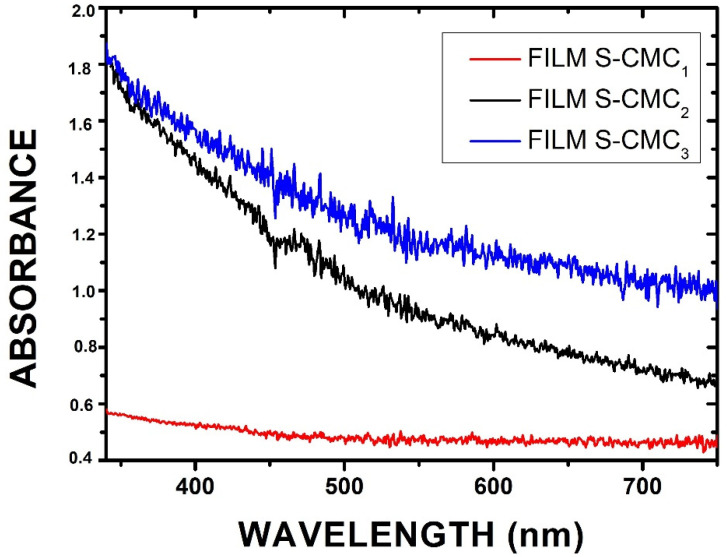
Absorbance spectra showing different behavior as a function of the concentration of CMC in the investigated films.

The optical properties of the obtained films were investigated by illuminating them with a focused white light beam. The spectroscopic system described in Section 2.2.3 was used to measure the absorbance (Abs) of samples, calculated as Abs=1−T−R, where T is the transmittance and R is the reflectance. Samples can scatter light, producing some light reflections. The absorbance of all samples was in the visible wavelength range (λ = 330–750 nm). In particular, the S-CMC_2_ and S-CMC_3_ films exhibited high absorbance values in the UV range (black and blue lines, respectively). The film S-CMC_1_ maintained the same trend, but a less intense signal (red line) was acquired. The spectral data are reported in the graphs of Figure 6.

Results confirmed the possible use of these films as a good alternative shield for food packaging. Since they presented high absorbance values in a large spectral range (UV-Vis-NIR), some of them could be a good absorptive layer. As reported in Section 2.2.1, the S-CMC_2_ and S-CMC_3_ films contain the highest quantities of CMC (0.75 g and 1 g, respectively). This explains the lowest absorbance values of the S-CMC_1_ film. The highest concentration (S-CMC_3_) represents the maximum absorption in the visible spectrum range, with a maximum in the UV-Vis range.

**Figure 7:**
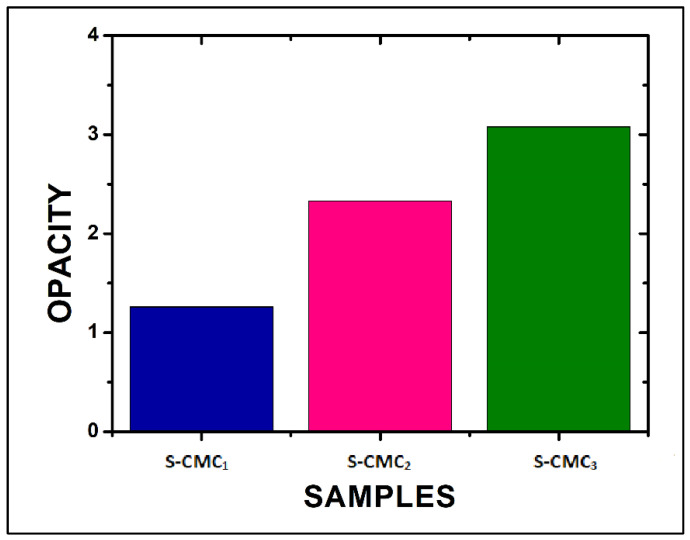
The opacity of the three films, evaluated at 600 nm by Equation (2).

The opacity, evaluated by Equation (2), increased as CMC concentration increased (Figure 7). The S-CMC_3_ film showed the maximum opacity, according to the absorbance measurements.

## 4. Conclusions

Gelification needs coalescence to occur, but aggregates should not exceed a critical size. If this happens, sedimentation or creaming phenomena prevail over gelification, reducing the film quality. Results suggested that when coalescence was more intense (increasing ΔT), particles had a size such that migration phenomena (sedimentation, creaming) prevailed over gelification. Consequently, phase separation was observed. It caused inhomogeneity zones, which can be seen in the films produced. Furthermore, the increased dispersion stability after the coalescence step—that is evident in the reduced TSI slope during time—provided information about the gelification degree.

The images acquired by the optical microscope showed the different morphologies of the three samples. S-CMC1 presented high roughness, whereas the S-CMC2 and S-CMC3 films are more homogenous, as confirmed by the analysis of the greyscale images. Besides, the absorbance of the S-CMC1 film was lower than the other two films. A film that presents a high value of absorbance represents a good packing able to cover and protect the compound from ultraviolet light (UV). The fact that the absorbance is not zero at other wavelengths ensures that the film should also be used to absorb the visible near-infrared (Vis-NIR) range.

A strict correlation between film-forming dispersions and optical properties of films was observed and discussed. Lower phase separation and higher stability in dispersions lead to higher quality of films, showing greater homogeneity and absorbance in the UV-Vis-NIR spectrum. The most promising film was obtained from the S-CMC3 dispersion.

Consequently, Turbiscan-based analysis of dispersions is useful as a preliminary step to forecast the final film quality. This analysis could be performed to investigate films from any polymer. Moreover, it is possible to understand the effectiveness of additives as cross-linking agents and plasticizers and their influence on dispersion stability and destabilization phenomena.

## Figures and Tables

**Table 1:** Composition and preparation method of dispersions.

Dispersion	Composition	Preparation
S	5 g starch 2 mL glycerol 1 g sodium hexametaphosphate 0.5 g citric acid 100 mL distilled water	5 min at room temperature + 30 min at 90 °C
CMC_1_	0.5 g CMC 75 mL distilled water	15 min at 75 °C
CMC_2_	0.75 g CMC 75 mL distilled water	15 min at 75 °C
CMC_3_	1 g CMC 75 mL distilled water	15 min at 75 °C
S-CMC_1_	S+CMC_1_	10 min at 75 °C
S-CMC_2_	S+CMC_2_	10 min at 75 °C
S-CMC_3_	S+CMC_3_	10 min at 75 °C

## Data Availability

The data presented in this study are available on request from the corresponding author.
